# A comparative study of orthokeratology and low-dose atropine for the treatment of anisomyopia in children

**DOI:** 10.1038/s41598-020-71142-3

**Published:** 2020-08-25

**Authors:** Wei-Shan Tsai, Jen-Hung Wang, Cheng-Jen Chiu

**Affiliations:** 1 Department of Ophthalmology, Hualien Tzu Chi Hospital, Buddhist Tzu Chi Medical Foundation, No. 707, Sec. 3, Chung-Yang Road, Hualien, 97002 Taiwan; 2 Department of Ophthalmology and Visual Science, Tzu Chi University, Hualien, Taiwan; 3 Department of Medical Research, Hualien Tzu Chi Hospital, Buddhist Tzu Chi Medical Foundation, Hualien, Taiwan

**Keywords:** Paediatric research, Vision disorders, Refractive errors, Eye abnormalities

## Abstract

Myopic anisometropia (anisomyopia) is a specific type of refractive error that may cause fusion impairment, asthenopia, and aniseikonia. It is sometimes severe enough to reduce the quality of life. Several studies have investigated the treatment effects of orthokeratology (Ortho-K) and topical atropine on anisomyopia control. However, no study has compared these two interventions simultaneously until now. The cohort of this retrospective study included 124 children with anisomyopia who were treated with binocular Ortho-K lenses, 0.01% atropine, or 0.05% atropine. After a 2-year follow-up, the inter-eye difference in axial length (AL) significantly decreased in the Ortho-K group (*P* = 0.015) and remained stable in the two atropine groups. When comparing the myopia control effect, the use of Ortho-K lenses resulted in an obviously smaller change in AL than the use of 0.01% and 0.05% atropine (*P* < 0.01). Ortho-K treatment may reduce the degree of anisomyopia and stabilise the progression of myopia. Hence, Ortho-K might be a better choice for anisomyopic children.

## Introduction

Anisometropia is a specific type of refractive error defined as a between-eye difference of ≥ 1.00 dioptre (D)^1–3^. According to the Ojai longitudinal study, the prevalence of anisometropia among 5–14-year-old children ranged between 0.9% and 2.4%^4^. In a more recent large population-based study, the prevalence of anisometropia among school-aged children was 5.3%^5^. The physician should be aware that the degree of anisomyopia may increase with the progression of myopia over time^.^^2,6–8^. A 3-year longitudinal study in Singapore also found that the frequency of anisometropia increased with time and each eye of anisometropic children exhibited a higher rate of myopia progression than the eyes of isometropic children^9^.

Orthokeratology (Ortho-K) lenses are highly oxygen-permeable, which can flatten the central cornea and temporarily decrease the degree of myopia. Ortho-K treatment has been heralded as the most effective non-pharmacological method of myopia control^10–12^. These reverse-geometry contact lenses can induce myopic defocus in peripheral retinae after corneal reshaping and thus achieve the goal of AL retarding effect and myopia management^13–17^. However, few studies have focused on the use of Ortho-K for anisomyopia regulation^16,18^. In our previous study, the magnitude of unilateral myopic anisometropia was reduced by monocular Ortho-K treatment, in which the inter-eye AL difference plummeted from 0.83 ± 0.45 mm at baseline to 0.59 ± 0.49 mm at 24 months (*P* = 0.039)^19^, thereby demonstrating that monocular Ortho-K lenses had effectively suppressed AL elongation of the myopic eyes in unilateral anisomyopic children.

Atropine is a muscarinic antagonist that was first used by Wells in 1900 to inhibit the progression of myopia^20,21^. High-dose (1%) atropine delivered in eye drops is the most efficacious pharmacological agent to prevent AL elongation^10^. Nevertheless, photophobia, decreased accommodation amplitude, and near vision difficulties are common ocular adverse effects in children^20^. The atropine for the treatment of childhood myopia (ATOM2) study found that atropine eye drops hampered myopia exacerbation and axial lengthening in children in a concentration-dependent manner^20^. However, an obvious rebound phenomenon was found with high-concentration atropine^20,22,23^. The dose related inability of accommodation was also reflected by the number of subjects needing photochromic lenses with reading addition in the study: 70% and 61% of the children treated with 0.5% and 0.1% atropine requested spectacles with reading addition, whereas only 6% of the children receiving 0.01% atropine treatment had the same demand^20^. They concluded that low-dose atropine eye drops had effectively slowed myopia advancement with fewer visual side effects than those with higher atropine concentrations^23^. In all anisometropia researches exploiting atropine, only one study^24^ demonstrated that monocular usage of 1% atropine eye drops had reduced the magnitude of myopic anisometropia from 1.82 ± 0.73 D at baseline to 0.47 ± 0.65 D after 9 months of treatment (*P* < 0.001) among 13 subjects with some tolerable aftereffects. However, no study has yet to evaluate the treatment effect of low-dose atropine on the control of anisomyopia.

Both Ortho-K and atropine eye drops are effective methods for myopia control, but few studies have compared them all together simultaneously, especially among anisomyopic children. Therefore, the primary aim of the present study was to compare the effect of Ortho-K lens versus low-dose (0.01% and 0.05%) atropine on the control of anisomyopia progression. The efficacy of myopia management of these treatment modalities were also evaluated.

## Materials and methods

### Patients

In this retrospective cohort study, the medical records of 1674 myopic children who underwent Ortho-K or topical atropine treatment at Hualien Tzu Chi Hospital (Hualien, Taiwan) from May 2012 to March 2019 were reviewed. The treatment modalities were chosen by the patients and their care-givers after receiving full explanations of the study goals. Among all, 124 patients with myopic anisometropia were identified and enrolled in our statistical analysis. By definition, their cycloplegic spherical equivalent refraction (SER) were -1.0 D or less in each eye with an inter-eye SER difference of 1.0 D or more. They were further categorised into three groups: binocular Ortho-K lenses (Group A), binocular 0.01% topical atropine with optimal spectacle correction (Group B), and binocular 0.05% topical atropine with optimal spectacle correction (Group C). This study adhered to the tenets of the Declaration of Helsinki and was approved by the Institutional Ethical Committee Review Board of Hualien Tzu-Chi Hospital. We had obtained written informed consent from all the patients including their parents if the children were under 18 years old before the recruitment. The inclusion and exclusion criteria were adapted from our previous publication^19^.

### Inclusion criteria

The minimal required age of the participants was 8 years old. Their refractive status was measured before and after cycloplegia. Only children who had fulfilled the three following criteria all at once would be considered recruiting into our study.

1. Best corrected visual acuity less then 0.00 log MAR (minimum angle of resolution) units
2. Cycloplegic SER of − 1.0 D or less in both eyes.
3. An inter-eye cycloplegic SER difference of 1.00 D or more.

### Exclusion criteria

1. Children with cycloplegic cylinder refraction of more than + 1.00 D or less than − 1.00 D.
2. History of binocular vision problems, including strabismus.
3. History of known ocular disorders, including media opacities, macular dysgenesis, optic nerve hypoplasia, perinatal brain injury, buphthalmos, and retinopathy of prematurity.
4. History of medication use that might have affected the refractive results.
5. Systemic or developmental problems that might have hindered refractive development.

In addition, children with history of other contact lenses use were not eligible for participation.

### The Ortho-K lens

The Ortho-K lenses used in this study were provided by Hiline Optical Co. (MacroVision Corporation, Taipei, Taiwan; Dk 85 × 10^−11^ (cm^3^O_2_/cm^2^S), 0.22-mm central thickness, and 10.6-mm diameter). To prevent from individual variation, all the fitting procedures were performed by the same consultant (C-J Chiu) with standardised method. Briefly, fluorescein stain was used to observe the centration of Ortho-K lenses. A good centration was defined as mild upward movement of the Ortho-K lens after blinking. Appropriate tightness was achieved by observing a thin annular layer of tears, trapped in the reverse curve, forming a bull’s eye configuration. All the fitting profile could be referred to our study published previously^11,19^. Children were required to wear the Ortho-K lenses every night for at least 8 consecutive hours. Patients were advised to seek an earlier visit if any ocular discomforts had been noticed.

### Atropine eye drops

Two low-dose atropine eye drops (0.01% atropine sulfate, 0.1 mg/1 mL, 0.5 mL/vial; 0.05% atropine sulfate, 0.5 mg/1 mL, 0.5 mL/vial) were provided by Aseptic Innovative Medicine Co., Ltd. (Taoyuan, Taiwan).

### Measurements and follow-up

At the first visit, screening tests were performed under full cycloplegia by instillation three drops of 1% tropicamide for 30 min. A serial of comprehensive ocular health examinations were set up, including cycloplegic autorefraction, corneal power, and keratometry, which were all measured by using an auto kerato-refractometer (KR-800; Topcon Europe Medical BV, Capelle aan den Ijssel, The Netherlands). Besides that, central corneal thickness was obtained from a computerised tonometer (CT-800; Topcon Europe Medical BV) and AL data was the results from an AL-scan optical biometer (Nidek Co., Ltd., Tokyo, Japan). Each parameter was determined by a mean value of three successive measurements. The routine followed-up time was set at one week, one month, and then every 3 months after lens delivery. During every visit, their unaided vision or spectacle-corrected visual acuity, SER error, corneal power, keratometry, and central corneal thickness were assessed, while the AL was measured at 1 month (baseline) and then every 6 months after lens fitting or atropine eye drop use. All the children in atropine groups commenced wearing optimal spectacles in the daytime since recruitment in order to fulfill the goal of best-corrected 6/6 vision. Furthermore, nine high myopic children in Ortho-K group were required to wear glasses for full correction owing to failure at achieving unaided visual acuity of 0.2 log MAR (6/9 on Snellen chart) during the daytime.

Two main outcomes were obtained and used as indicators of treatment effect: the inter-eye AL difference and the change in AL (ΔAL). Their definition could be referred to Eqs. (1) and (2) listed below:

1 $$  {\text{Inter}} - {\text{eye}}\;{\text{AL}}\;{\text{ difference}} = (A - B){\text{ }} $$

A: AL of the higher myopic eye, B: AL of the lower myopic eye.

2 $$ \Delta {\text{AL}} = ({\text{A}} - {\text{B}})$$

A: AL at different follow-up periods, B: AL at baseline.

### Statistical analysis

When comparing the baseline demographics among the three treatment groups, data is presented as frequencies, proportions, means ± standard deviations (SD), or median (minimum, maximum), according to the characteristics of each item. Some statistical tests were performed to compare the differences between groups. The chi-squared test or Fisher’s exact test was used to evaluate the association between two categorical variables. One-way analysis of variance or Kruskal–Wallis test was used to test if there were significant differences between groups. Post-hoc test was performed via Bonferroni correction. Trend analysis was adopted to evaluate the time effect on inter-eye AL difference and ΔAL. After restricting the data collection to the right eyes, the associations between risk factors and ΔAL during the follow-up period were evaluated by a generalised estimating equation (GEE). Covariates, such as treatment, intervention age, gender, follow-up time, and baseline AL, were included in the GEE model. Statistically significant differences were defined as *P* < 0.05. All statistical analyses were performed using SPSS Statistics for Window, version 17.0 (SPSS Inc., Chicago, IL, USA).

## Results

Table 1 summarises the demographic data of our patients. In brief, we analysed a total number of 124 children with myopic anisometropia, and separated them into different groups according to their treatment modalities (Group A: binocular Ortho-K treatment, group B: binocular 0.01% atropine with spectacle correction, group C: binocular 0.05% atropine with spectacle correction). The average intervention age was 11.99 ± 2.19 years, and the mean follow-up duration was 1.43 ± 0.59 years. In terms of the more myopic eyes, the Ortho-K treatment group had a longer initial AL and higher initial spherical equivalent (SE) compared to 0.01% atropine and 0.05% atropine group (*P* < 0.001). This condition was also observed in the less myopic eyes (*P* < 0.001). There was no significant difference in gender, initial inter-eye spherical equivalent difference (median: 1.25 D), and initial inter-eye axial length difference (average 0.46 ± 0.26 mm) among these three groups.

**Table 1:** Demographics (N = 124).

Variable	Group A Ortho-K	Group B 0.01%A	Group C 0.05%A	Total	P-value	Post-hoc
N	52	36	36	124		
**Age Group**					0.235	
< 12 y/o	22(42.3%)	20(55.6%)	13(36.1%)	55(44.4%)		
≧12 y/o	30(57.7%)	16(44.4%)	23(63.9%)	69(55.6%)		
**Gender**					0.552	
Male	21(40.4%)	18(50.0%)	18(50.0%)	57(46.0%)		
Female	31(59.6%)	18(50.0%)	18(50.0%)	67(54.0%)		
Inter-eye SE difference (D)^&^	1.27(1.00, 3.38)	1.25(1.00, 2.50)	1.25(1.00, 2.75)	1.25(1.00, 3.38)	0.983	
Inter-eye AL difference (mm)	0.53 ± 0.29	0.41 ± 0.25	0.42 ± 0.20	0.46 ± 0.26	0.069	
**More myopic eye**						
Initial AL (mm)	25.33 ± 1.19	24.71 ± 1.29	24.35 ± 0.82	24.82 ± 1.13	< 0.001*	A > B, C
Initial SE (D)	− 5.27 ± 2.31	− 3.36 ± 1.73	− 3.12 ± 1.35	− 4.09 ± 2.14	< 0.001*	A < B, C
Initial K1 (D)	43.00 ± 1.31	42.93 ± 1.31	42.83 ± 1.29	42.93 ± 1.30	0.832	
Initial K2 (D)	44.33 ± 1.45	44.02 ± 1.54	43.78 ± 1.40	44.08 ± 1.47	0.219	
**Less myopic eye**						
Initial AL (mm)	24.84 ± 1.18	24.00 ± 0.64	23.87 ± 0.65	24.38 ± 1.11	< 0.001*	A > B, C
Initial SE (D)	− 3.77 ± 2.19	− 1.88 ± 1.66	− 1.66 ± 1.38	− 2.61 ± 2.07	< 0.001*	A < B, C
Initial K1 (D)	42.92 ± 1.34	42.92 ± 1.47	42.80 ± 1.24	42.89 ± 1.34	0.901	
Initial K2 (D)	44.36 ± 1.44	44.08 ± 1.47	43.85 ± 1.48	44.13 ± 1.47	0.266	
Follow-up Time (years)	1.47 ± 0.61	1.51 ± 0.57	1.29 ± 0.59	1.43 ± 0.59	0.232	

Data are presented as n(%) or mean ± standard deviation or ^&^median (minimum, maximum).

*p-value < 0.05 was considered statistically significant after test.

A = atropine, AL = axial length, D = dioptre, K = keratometry, K1 = the refraction measured on the flattest curve of the cornea, K2 = the refraction measured on the deepest curve of the cornea, mm = millimetre, Ortho-K = orthokeratology, SE = spherical equivalent.

The calculation of inter-eye AL difference during the study period was presented in Table 2. In Ortho-K treatment group, the inter-eye AL difference plummeted significantly from 0.53 ± 0.29 mm at the baseline to 0.38 ± 0.29 mm at 24 months (*P* = 0.015). There was a slight decrease in the 0.05% atropine group, from 0.42 ± 0.20 mm initially to 0.34 ± 0.19 mm in the end (*P* = 0.111). Conversely, no significance was found in the inter-eye AL difference of the 0.01% atropine group, which started with 0.41 ± 0.25 mm at the beginning and remained at a similar length of 0.42 ± 0.27 mm finally (*P* = 0.981).

**Table 2:** Trend of inter-eye axial length difference among three groups.

Group	Item	Follow-up Time(Months)					P for trend
		Baseline	6 M	12 M	18 M	24 M	
	N	52	52	41	35	25	0.015*
Group A Ortho-K	AL of more myopic eyes (mm)	25.33 ± 1.19	25.35 ± 1.12	25.31 ± 1.12	25.39 ± 1.06	25.68 ± 0.95	
	AL of less myopic eyes (mm)	24.84 ± 1.18	24.88 ± 1.16	24.90 ± 1.20	24.95 ± 1.12	25.30 ± 1.01	
	Inter-eye AL difference (mm)	0.53 ± 0.29	0.50 ± 0.29	0.43 ± 0.26	0.44 ± 0.27	0.38 ± 0.29	
Group B 0.01%A	N	36	36	31	24	18	0.981
	AL of more myopic eyes (mm)	24.71 ± 1.29	24.80 ± 1.08	24.84 ± 1.24	25.27 ± 1.37	25.57 ± 1.53	
	AL of less myopic eyes (mm)	24.00 ± 0.64	24.38 ± 1.07	24.39 ± 1.20	24.86 ± 1.35	25.15 ± 1.47	
	Inter-eye AL difference (mm)	0.41 ± 0.25	0.43 ± 0.25	0.44 ± 0.25	0.41 ± 0.26	0.42 ± 0.27	
Group C 0.05%A	N	36	36	29	15	13	0.111
	AL of more myopic eyes (mm)	24.35 ± 0.82	24.47 ± 0.81	24.62 ± 0.82	24.82 ± 0.78	25.00 ± 0.82	
	AL of less myopic eyes (mm)	23.87 ± 0.65	24.13 ± 0.81	24.31 ± 0.81	24.56 ± 0.72	24.75 ± 0.76	
	Inter-eye AL difference (mm)	0.42 ± 0.20	0.43 ± 0.21	0.42 ± 0.23	0.34 ± 0.21	0.34 ± 0.19	

Data are presented as n or mean ± standard deviation.

*AL* axial length, *A* atropine, *M* month, *Ortho-K* orthokeratology.

Table 3 evaluates several factors and their association with the AL changes. We used gender, intervention age, baseline AL, and the different treatment modalities as variable factors to calculate their correlation with the AL change by the GEE model. The results showed that intervention age, gender, and baseline AL exhibited no effect on AL change (*P* = 0.104, 0.624, and 0.658, respectively). Among these groups, Ortho-K treatment had a significant effect on AL change when comparing to 0.05% atropine group (β = -0.135, *P* = 0.010), and there was no difference between 0.01% atropine and 0.05% atropine treatment group (β = 0.110, *P* = 0.062) over 2 years follow-up.

**Table 3:** Generalized estimating equations (GEE) model predicting AL change of right eyes over 2 years follow-up. (N = 124).

Predictor	β	95% CI	p value
Intercept	0.510	(− 0.725, 1.745)	0.418
**Group**			
Ortho-K versus 0.05%A	− 0.135	(− 0.237, − 0.033)	0.010*
0.01%A versus 0.05%A	0.110	(− 0.006, 0.225)	0.062
Intervention age	− 0.015	(− 0.033, 0.003)	0.104
Gender (male vs. female)	− 0.025	(− 0.126, 0.076)	0.624
Time	0.132	(0.110, 0.154)	< 0.001*
Baseline AL	− 0.012	(− 0.067, 0.043)	0.658

*AL* axial length, *A* atropine, *Ortho-K* orthokeratology.

*p-value < 0.05 was considered statistically significant after test.

**Figure 1:**
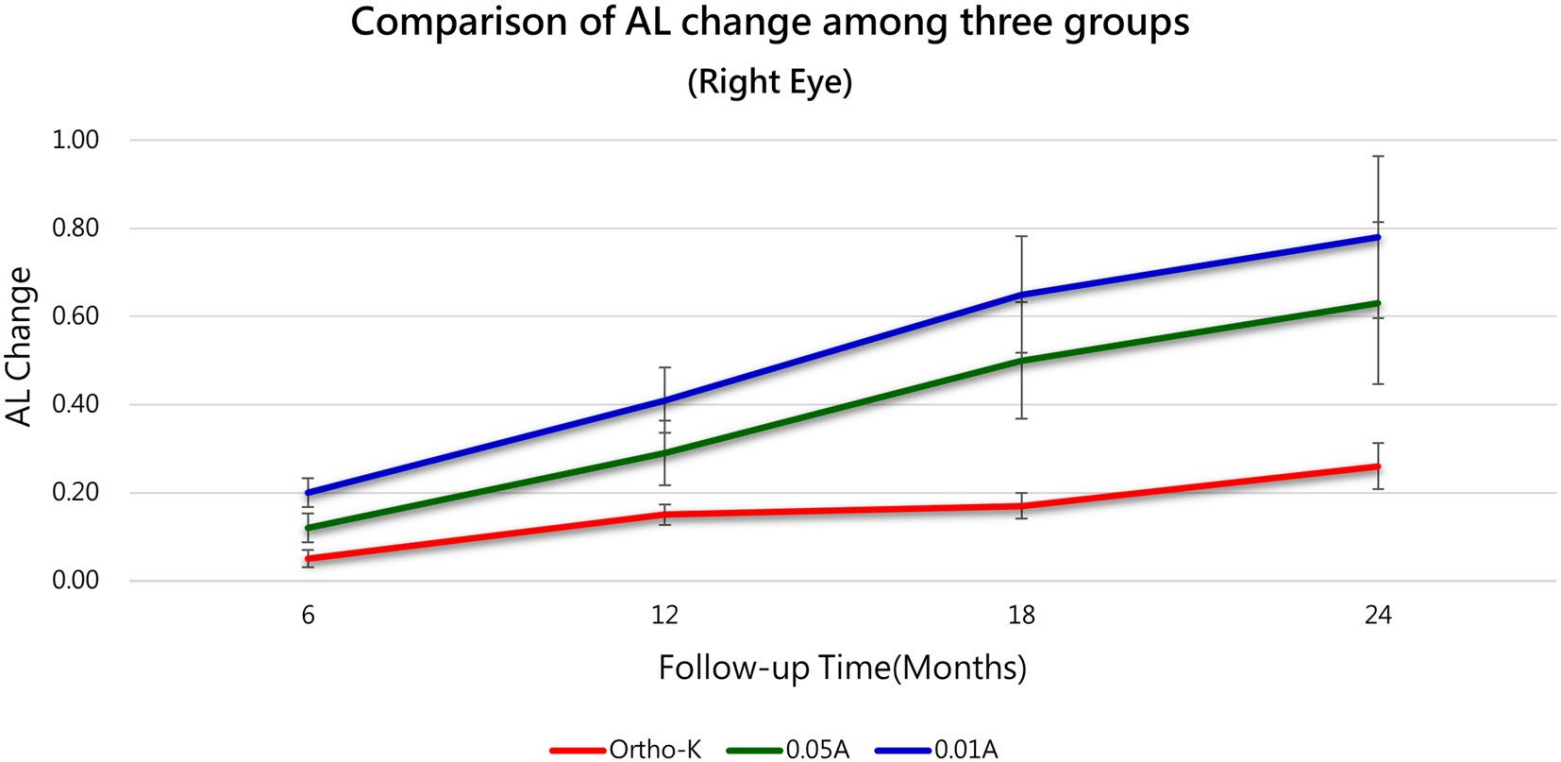
Monocular change in axial length (ΔAL) after intervention with three treatment modalities. Though an increase in AL was observed in all treatment groups with time (*P* for trend = 0.001, < 0.001, < 0.001 for the Ortho-K, 0.01% atropine, and 0.05% atropine groups, respectively), Ortho-K group showed an obvious suppression of AL length growth compared to the other two methods (Between-group *P*-value < 0.001 at any time point). Abbreviations: A = atropine, AL = axial length, Ortho-K = orthokeratology.

Table 4 further depicts the AL change of right eyes between the baseline and every follow-up time in each group, which could be the representative of AL slowing effect by different treatment modalities. A steady growth of AL was observed in all treatment groups with time trend (*P* for trend = 0.001, < 0.001, < 0.001 in Ortho-K, 0.01% atropine, and 0.05% atropine group, respectively). But if we compared these three therapies all together at different cross-sectional time, the Ortho-K treatment group disclosed an exceptionally stronger AL stabilising effect than any other two groups (Between-group *P*-value < 0.001 and 0.001). At 18-month and 2-year follow-up, 0.05% atropine group demonstrated a significantly smaller AL change than 0.01% group (Between-group *P*-value < 0.001) as shown in Table 4 and Fig. 1.

**Table 4:** Comparison of AL change of right eyes among three groups.

Side	Group	Item	Follow-up Time (Months)				P for trend
			6 M	12 M	18 M	24 M	
Right	Group A	N	52	41	35	25	0.001*
	Ortho-K	AL Change	0.05 ± 0.14	0.15 ± 0.28	0.17 ± 0.26	0.25 ± 0.32	
	Group B	N	36	31	24	18	< 0.001*
	0.01%A	AL Change	0.20 ± 0.20	0.41 ± 0.32	0.65 ± 0.41	0.78 ± 0.40	
	Group C	N	36	29	15	13	< 0.001*
	0.05%A	AL Change	0.12 ± 0.15	0.29 ± 0.23	0.50 ± 0.33	0.63 ± 0.38	
	Between-group	P-value	< 0.001*	0.001*	< 0.001*	< 0.001*	
	Post-hoc		A < B	A < B	A < C < B	A < C < B	

Data are presented as n or mean ± standard deviation.

*AL* axial length, *AL Change* AL at different follow-up time—AL at baseline, *A* atropine, *M* month, *Ortho-K* orthokeratology.

During the study period, there were no reports of severe treatment-related events including corneal warpage or infectious keratitis in the Ortho-K treatment group. Also, there were no allergy reactions or intolerable photophobia in two atropine groups.

## Discussion

Anisometropia is a special form of refractive error with a prevalence that tends to increase with age. For example, Deng et al. reported that the prevalence of anisometropia had trebled from 1.96% in children aged 6 months to 5.77% in adolescents aged 12–15^6^. Without proper treatment, the more ametropic eye of a high anisomyopic patient (inter-ocular difference > 4D) will experience a rapid growth, resulting in an even greater extension of anisomyopia and a higher degree of myopia^3^. Recent studies have focused particularly more on this specific anisomyopic children by comparing the experimental eye with the patient’s fellow eye as a naturally matched group^18^. Both Ortho-K and topical atropine have been reported to have positive effects on the control of anisomyopia^18,19,24^. Therefore, the aim of the present study was to further compare the treatment effect of Ortho-K to that of different atropine concentrations in anisomyopic children.

In this study, both eyes of anisomyopic children were treated either with Ortho-K lenses, 0.01% atropine, or 0.05% atropine. Their AL were evaluated every 6 months. We found that there was a significant decrease in the inter-eye AL difference in Ortho-K group (*P* for trend = 0.015) compared to low-dose atropine group, which indicated that Ortho-K was a superior treatment for anisometropic patients. In addition, the longitudinal ΔAL of right eyes was more inclined to level off in Ortho-K group than in atropine groups, therefore, it would be more advantageous to treat myopia with Ortho-K.

With regard to the use of Ortho-K as treatment modality for anisomyopia, the study results showed that 2 years of bilateral Ortho-K therapy had significantly ameliorated the magnitude of anisomyopia by reducing 0.15 mm in inter-eye AL difference. Recently, Zhang and Chen reported a significant slump (0.16 ± 0.04 mm) in inter-ocular AL difference among anisomyopic children after 2 years of wearing binocular Ortho-K lenses^18^. Although the underlying mechanism remained unclear, the authors proposed that their finding was a result of greater mid-peripheral corneal re-shaping in the higher myopic eyes. This produced a stronger peripheral retinal defocus and a more effective myopic control^18,25,26^, and hence, the magnitude of anisometropia was diminished sequentially. In Zhang and Chen’s study, the mean SE of the more myopic eyes was − 5.00 D (which fit the criteria of high myopia^27^), whilst the average degree of anisometropia was 1.75 D and the inter-eye AL difference was 0.72 mm^18^. In contrast, the representative SE of the higher myopic eyes in our study was comparably milder (− 4.09 D). In line with that, the average degree of anisometropia was also smaller (1.50 D) with a shorter inter-eye AL difference (0.46 mm). The comparison revealed that Ortho-K could deplete the extent of anisomyopia in patients with an even lower level of myopia and anisometropia. Our study also reinforced the hypothesis of enhanced peripheral myopic defocus in high myopes, as the more myopic eyes in Ortho-K group disclosed a less AL elongation in Table 2.This phenomenon has been widely discussed by another two latest studies including Fu et al.^28^ and Zhong et al.^29^ The latter has underpinned our deduction by their findings on a significant plunge in myopic progression in the greater myopic eyes compared to the less myopic fellow eyes (-0.84 ± 0.63 D versus −1.21 ± 0.89 D, *P* < 0.001)^29^. Though Fu et al. only yielded affirmative results from unilateral myopic children wearing monocular Ortho-K lenses rather than all the anisomyopes in their study, they attributed this to the smaller anisometropic value on the part of binocular Ortho-K group and fully supported the theory of peripheral myopic defocus^28^.

To date, only one study by Lin et al.^24^ has investigated the treatment outcomes of monocular 1% atropine on myopic anisometropia. The result of that study showed a reduced level of anisometropia from 1.82 ± 0.73 D to 0.47 ± 0.65 D with a significant P-value (*P* < 0.001) after an average of 11.5-month treatment. By implication, whilst the atropine treated eye experienced a smaller amount of elongation, the non-treated eye kept lengthening as a natural course. The author then surmised that the consequence was emanated from an unequal axial length growth between both eyes. With this similar explanation, we proposed that in our binocular treatment study, atropine eye drops had generated an impartial inhibitory effect on both eyes, which resulted in an insignificant change in inter-eye AL difference. Based on rigorous literature review, we concluded that the present study might be the first study to evaluate the treatment effect of bilateral topical atropine administration designed especially on anisomyopia control.

To find the answer regarding the therapeutic choice for myopia, most studies enrolled matched children wearing spectacles in comparison with the Ortho-K group^30^. Until now, only one study by Lin et al^31^. has compared the effect of Ortho-K versus topical atropine on the control of myopia. In that study, axial length were checked every year after discontinuing Ortho-K lenses for 3 weeks, and the linear regression analysis revealed a significant difference in AL growth between the Ortho-K lens (0.28 mm) and 0.125% atropine (0.37 mm) group^31^. Since low-dose atropine is an emerging therapy popularly accepted for preventing myopia progression^23^, we compared the effect of myopia control between Ortho-K and low-dose atropine by calculating AL lengthening every 6 months in three different groups. Our results disclosed greater suppression of AL elongation in the Ortho-K group as compared to the 0.01% and 0.05% atropine groups at different time points (between-group *P* < 0.001). In addition, there was no information about the amount of anisometropia of Lin’s subjects. Contrarily, our study offered clinicians a clue that Ortho-K was more beneficial to treat anisometropia.

Both Ortho-K and atropine are effective for myopia control but through different pathways. The most well-known mechanism of Ortho-K is the generation of peripheral myopic defocus that stabilises axial eye elongation and attenuates myopia progression^11^. Though it is not yet completely understood, several studies have postulated that the effect of atropine involves the M1/M4 receptors in retinae^32^ or the dopamine signaling pathways^33,34^, eventually leading to scleral remodelling^32,35^. Since a higher concentration of atropine has a greater inhibitory but a more rebounding effect on AL growth after cessation^20,36^, low-dose atropine (0.01% and 0.05%) were used in the present study because of the acceptable myopia control effect and fewer adverse events^36,37^. As compared to a placebo, low-dose atropine eye drops (0.05%, 0.025%, and 0.01%) reportedly reduced myopia progression in a concentration-dependent manner^23^. Based on the observed results, we conjectured that the peripheral myopic defocus effect obtained from the Ortho-K lenses might be more consistent and stable than that with the use of low-dose atropine. Furthermore, there might be a saturation limit between low- concentration atropine and their associated receptors. The above-mentioned factors might contribute to the superior stabilisation effect of Ortho-K to low-dose atropine eye drops on ΔAL.

There were some limitations of our study. First of all, no sham treatment group was established for comparisons. The second limitation was the retrospective nature of the study. Thirdly, the statistical analysis might have been influenced by the different follow-up periods; therefore, a GEE model was adopted. Finally, a 2-year follow-up may be insufficient to reveal the complete and lasting effect of all modalities for treatment of anisometropia and myopia control. This could possibly explain the reason why there was a tendency to show the difference, but not a statistically significant one, in ΔAL between the two atropine groups (*P* = 0.062, Table 3. Therefore, further studies with long-term follow-up are needed to verify these results.

## Conclusions

This is the first study to compare the therapeutic methods of Ortho-K lenses versus 0.01% and 0.05% atropine for the treatment of anisomyopia in children. During the 2-year treatment period with Ortho-K, the more myopic eyes had experienced less AL elongation than the less myopic eyes in anisomyopic children, and thus the inter-eye difference in AL was substantially decreased (*P* < 0.05). Low-dose atropine (0.01% and 0.05%) could maintain the inter-eye AL difference and retard the progression of anisometropia. On the other hand, all three approaches exhibited a stabilising effect on ΔAL with time. Among all treatment modalities, Ortho-K achieved significantly better control of myopia than 0.01% and 0.05% atropine. Collectively, these findings demonstrate that Ortho-K treatment can reduce the magnitude of anisomyopia and slow the progression of myopia. Hence, Ortho-K might be a better choice for treatment of anisomyopia in children, for it not only attains optimum myopia control in both eyes, but lessons the degree of anisomyopia. Nonetheless, further studies are required to validate the results of the present study.

## References

[1] Karimian F, Ownagh V, Amiri M, Tabatabaee S, Dadbin N (2017). Stereoacuity after wavefront-guided photorefractive keratectomy in anisometropia. J. Ophthalmic Vis. Res..

[2] Vincent SJ, Collins MJ, Read SA, Carney LG (2014). Myopic anisometropia: ocular characteristics and aetiological considerations. Clin. Exp. Optometry.

[3] Zedan RH, El-Fayoumi D, Awadein A (2017). Progression of high anisometropia in children. J. Pediatr. Ophthalmol. Strabismus.

[4] Hirsch MJ (1967). Anisometropia: a preliminary report of the Ojai Longitudinal Study. Am. J. Optom. Arch. Am. Acad. Optom..

[5] Lee C-W (2017). Prevalence and association of refractive anisometropia with near work habits among young schoolchildren: The evidence from a population-based study. PLoS ONE.

[6] Deng L, Gwiazda JE (2012). Anisometropia in children from infancy to 15 years. Investig. Ophthalmol. Vis. Sci..

[7] Hu YY (2016). Prevalence and associations of anisometropia in children. Investig. Ophthalmol. Vis. Sci..

[8] Pärssinen O, Kauppinen M (2017). Anisometropia of spherical equivalent and astigmatism among myopes: a 23-year follow-up study of prevalence and changes from childhood to adulthood. Acta Ophthalmol..

[9] Tong L, Chan YH, Gazzard G, Tan D, Saw SM (2006). Longitudinal study of anisometropia in Singaporean school children. Investig. Ophthalmol. Vis. Sci..

[10] Huang J (2016). Efficacy comparison of 16 interventions for myopia control in children a network meta-analysis. Ophthalmology.

[11] Lee YC, Wang JH, Chiu CJ (2017). Effect of Orthokeratology on myopia progression: twelve-year results of a retrospective cohort study. BMC Ophthalmol..

[12] Walline J, Smith M (2015). Controlling myopia progression in children and adolescents. Adolesc. Health. Med. Ther..

[13] Chen C, Cheung SW, Cho P (2013). Myopia control using toric orthokeratology (TO-SEE study). Investig. Ophthalmol. Vis. Sci..

[14] Cho P, Cheung SW (2012). Retardation of myopia in orthokeratology (ROMIO) study: a 2-year randomized clinical trial. Investig. Ophthalmol. Vis. Sci..

[15] Hiraoka T, Kakita T, Okamoto F, Takahashi H, Oshika T (2012). Long-term effect of overnight orthokeratology on axial length elongation in childhood myopia: a 5-year follow-up study. Investig. Ophthalmol. Vis. Sci..

[16] Lum E (2018). Progressive anisometropia and orthokeratology: a case report. Clin. Exp. Optometry.

[17] Walline JJ, Jones LA, Sinnott LT (2009). Corneal reshaping and myopia progression. J Ophthalmol.

[18] Zhang Y, Chen Y (2019). Effect of orthokeratology on axial length elongation in anisomyopic children. Optom. Vis. Sci..

[19] Tsai WS, Wang JH, Lee YC, Chiu CJ (2019). Assessing the change of anisometropia in unilateral myopic children receiving monocular orthokeratology treatment. J. Formos. Med. Assoc..

[20] Chia A (2012). Atropine for the treatment of childhood myopia: safety and efficacy of 0.5%, 0.1%, and 0.01% doses (atropine for the treatment of myopia 2). OPHTHA.

[21] Chia A, Lu QS, Tan D (2016). Five-year clinical trial on atropine for the treatment of myopia 2 myopia control with atropine 0.01% eyedrops. Ophthalmology.

[22] Tong L (2009). Atropine for the treatment of childhood myopia: effect on myopia progression after cessation of atropine. OPHTHA.

[23] Yam JC (2019). Low-concentration atropine for myopia progression (LAMP) study. Ophthalmology.

[24] Lixia L (2013). Treatment outcomes of myopic anisometropia with 1% atropine: a pilot study. Optom. Vis. Sci..

[25] Charm J, Cho P (2013). High myopia-partial reduction ortho-k: A 2-year randomized study. Optom. Vis. Sci..

[26] Kakita T, Hiraoka T, Oshika T (2011). Influence of overnight orthokeratology on axial elongation in childhood myopia. Investig. Ophthalmol. Vis. Sci..

[27] Bazzazi N, Akbarzadeh S, Yavarikia M, Poorolajal J, Fouladi DF (2017). High myopia and diabetic retinopathy: a contralateral eye study in diabetic patients with high myopic anisometropia. Retina.

[28] Fu A-C (2020). Effects of orthokeratology lens on axial length elongation in unilateral myopia and bilateral myopia with anisometropia children. Contact Lens Anterior Eye.

[29] Zhong Y, Li K, Wu Q, Liu F (2020). Orthokeratology lens for management of myopia in anisometropic children: a contralateral study. Contact Lens Anterior Eye.

[30] VanderVeen DK (2019). Use of orthokeratology for the prevention of myopic progression in children: a report by the american academy of ophthalmology. Ophthalmology.

[31] Lin HJ (2014). Overnight orthokeratology is comparable with atropine in controlling myopia. BMC Ophthalmol..

[32] Wu PC (2019). Update in myopia and treatment strategy of atropine use in myopia control. Eye (Basingstoke).

[33] Schmid KL, Wildsoet CF (2004). Inhibitory effects of apomorphine and atropine and their combination on myopia in chicks. Optom. Vis. Sci..

[34] Zhou X, Pardue MT, Iuvone PM, Qu J (2017). Dopamine signaling and myopia development: what are the key challenges. Prog. Retin. Eye Res..

[35] Mcbrien NA, Gentle A (2003). Role of the sclera in the development and pathological complications of myopia. Prog. Retinal Eye Res..

[36] Chia A (2014). Atropine for the Treatment of Childhood Myopia: Changes after Stopping Atropine 0.01%, 0.1% and 0.5%. Ophthalmol.

[37] Wu P-C, Yang Y-H, Fang P-C (2011). The long-term results of using low-concentration atropine eye drops for controlling myopia progression in schoolchildren. J. Ocul. Pharmacol. Ther..

